# Negative Gossip Decreases Targets’ Organizational Citizenship Behavior by Decreasing Social Inclusion. A Multi-Method Approach

**DOI:** 10.1177/1059601120986876

**Published:** 2021-01-26

**Authors:** Elena Martinescu, Wiebren Jansen, Bianca Beersma

**Affiliations:** 1 1190 Vrije Universiteit Amsterdam, Amsterdam, Netherlands; 2 8125 Utrecht University, Utrecht, Netherlands

**Keywords:** negative gossip, gossip target, social inclusion, organizational citizenship behavior, cooperation

## Abstract

Ample experimental evidence shows that negative gossip fosters cooperation in groups by increasing individuals’ reputational concerns. However, recent field studies showed that negative gossip decreases organizational citizenship behavior (OCB) among its targets (i.e., people whom gossip is about). Bridging these findings, we study the role of social inclusion in explaining how negative gossip affects targets’ engagement in OCB. Based on social exchange theory, we predict that targets of negative gossip experience low social inclusion. In turn, we propose that low social inclusion leads to low OCB of gossip targets. Results of three studies, a correlational study (*N* = 563), a laboratory experiment (*N* = 85), and an online scenario experiment (*N* = 597), showed that being the target of negative gossip reduced social inclusion and indirectly decreased OCBs. Our multi-method approach bridges findings from research conducted in organizations and in laboratory experiments and offers a more nuanced understanding of the effects of negative gossip on targets’ behavior. We show that due to its detrimental effect on targets’ social inclusion, negative gossip may not be as effective for enabling sustainable cooperation as experimental studies claim it to be.

## Introduction

Gossip, or talk about others who are not present, which is often informal and evaluative, is pervasive in daily interactions (Dunbar, 2004; Dunbar, Marriott, & Duncan, 1997; Emler, 1994; Foster 2004). Gossip targets (i.e., the people whom gossip is about) are absent when gossip is shared and have limited access and control over it. Gossip can be positive or negative, and research shows that positive and negative gossip co-occur frequently (Kniffin & Wilson, 2005). In this article, we focus on negative gossip, which transmits messages with negative content about its targets and is particularly problematic for them, as negative information is easier to recall and is more likely to influence perceptions about targets than positive or neutral information (Baumeister, Bratslavsky, Finkenauer, & Vohs, 2001). Negative gossip is likely to harm targets’ reputation because it exposes their flaws or norm-breaking behavior to others (Beersma & Van Kleef, 2011; Feinberg, Willer, Stellar, & Keltner, 2012).

As such, people who become targets of negative gossip may choose to behave cooperatively at key moments, to avoid further spread of negative gossip and to protect their reputation. Indeed, experimental research has shown that under gossip threat, people allocate more of their resources to others in economic games (Beersma & Van Kleef, 2011; Piazza & Bering, 2008; Wu, Balliet, & Van Lange, 2016a) and are more generous if gossip about them can easily reach others (Wu, Balliet, & Van Lange, 2016b). These findings have been used to argue that negative gossip elicits desirable behaviors from (potential) gossip targets, who fear for their reputation. For example, Wu et al. (2016a) proposed that “gossip and reputation systems could have a sustainable effect on cooperation and potentially cultivate voluntary cooperation” (p. 5).

This implies that in real-life settings, such as the workplace, negative gossip should lead gossip targets to behave cooperatively in order to counteract the reputational threat posed by negative gossip. An important way in which cooperation is manifested in organizations is when employees engage in organizational citizenship behaviors (OCBs). OCBs represent extra role (i.e., not prescribed by one’s work role), discretionary, and unmonitored contributions by group members (Organ & Ryan, 1995). OCBs are essential for the proper functioning of work groups, as these rely on contributions from their members, beyond those specified in formal agreements (Nielsen, Hrivnak, & Shaw, 2009). Engaging in OCBs fosters group performance by enhancing coworker productivity (Podsakoff & MacKenzie, 1997) and by reducing the supervisory workload of group leaders (Podsakoff, MacKenzie, Paine, & Bachrach, 2000). Based on the results of experimental research discussed above, one would expect negative gossip to have a beneficial and sustainable effect on OCB in organizational settings. Because OCB may be witnessed by one’s colleagues and supervisors, this behavior could help (re-)build one’s reputation after being targeted by negative gossip. Indeed, a recent study using organizational samples showed that targets of negative gossip predisposed to high self-monitoring may use OCB as a reputation management tactic (Xie, Huang, Wang, & Shen, 2019).

However, the idea that negative gossip would lead to higher OCB of targets stands in stark contrast to survey research showing that negative gossip is negatively related to targets’ OCB. Specifically, perceiving negative workplace gossip was found to be detrimental for targets’ work-related self-concept (defined as perceived insider status and organizational-based self-esteem), which, in turn, is related to lower OCB (Kong, 2018; Wu, Birtch, Chiang, & Zhang, 2018a). Similarly, negative gossip was found to relate to lower OCB by targets, due to damaging effects on targets’ identification with their organization (Ye, Zhu, Deng, & Mu, 2019). Other research demonstrated that negative gossip was emotionally exhausting for targeted individuals, who became less able to engage in proactive behaviors that benefit the organization as a result of it (Wu, Kwan, Wu, & Ma, 2018b). Overall, organizational research points to a negative relationship between negative gossip and OCB of targets, due to the effects of negative gossip on their negative self-views and emotions.

The inconsistent conclusions that can be drawn from experimental and survey studies in organizations are problematic because they stand in the way of theoretical integration and of providing clear directions to organizations on how negative gossip should be viewed and managed. In this article, we aim to bridge these lines of research, by investigating the effect of negative gossip on targets’ citizenship behavior in both field and experimental settings, and provide a more comprehensive understanding of mechanisms at work.

We focus on a mediating mechanism that has not been examined in previous research, but which is likely to play an important role in explaining the effect of negative gossip on targets’ OCB: social inclusion. Social inclusion is a concept rooted in optimal distinctiveness theory (ODT, Brewer, 1991) and self-determination theory (SDT, Deci & Ryan, 2000) and represents the extent to which people feel that a group satisfies their needs for belongingness and authenticity. The need for belongingness is satisfied when people form and maintain strong and stable relationships with others. The need for authenticity is satisfied when people are allowed and encouraged to behave in accordance with their integrated sense of self (Jansen, Otten, Van der Zee, & Jans, 2014). Social inclusion is an important determinant of individuals’ behavior within the group. People who feel socially included are likely to act in ways that benefit the group (c.f. Ellemers & Jetten, 2013; Jansen, Meeussen, Jetten, & Ellemers, 2020), such as engaging in OCB. Because people experiencing high social inclusion perceive a strong bond with their group (Jansen et al., 2014), they may choose to act in the group’s best interest, without external monitoring or enforcement, in line with a reciprocity principle (Blau, 1964). However, targets of negative gossip may perceive that group members disregard their needs for belongingness and authenticity because negative gossip may threaten targets’ reputation and relationships with others and denies them the chance to present their perspective on issues that concern them, respectively. As such, negative gossip may be detrimental for targets’ behavior aimed at benefitting the group.

We propose that individuals’ experiences of being targeted by negative gossip harm their social inclusion in work groups, and subsequently their engagement in OCB. Earlier experimental studies (Beersma & Van Kleef, 2011; Piazza & Bering, 2008; Wu et al., 2016a) have not examined variables that reflect the way in which gossip targets perceive their social relationship with the group as a result of gossip. As such, they have overlooked the possibility that by decreasing targets’ inclusion, negative gossip may be detrimental for a key resource in organizations—employees’ citizenship behavior—and may create a lot of damage “under the surface.” Therefore, by using different methodologies, samples, and measures, we investigate how people experience their inclusion in groups where others gossip negatively about them, and how this experience affects their citizenship behavior. We thus contribute to the gossip and OCB literatures by providing a broader understanding of the consequences of negative gossip for targets’ OCB, and the mechanisms that bring about these consequences.

## Theoretical Background and Hypotheses

### Social Exchange Relationships of Negative Gossip Targets

According to social exchange theory (SET), people interact to exchange socially valued resources, such as information, affection, status, goods, or services (Cropanzano & Mitchell, 2005). The exchange is guided by a set of norms, of which reciprocity is most important. People in reciprocal exchange relationships are interdependent, meaning that their actions are contingent on the actions of the other person(s), and the typical response is “repayment in kind” (Blau, 1964). Moreover, people who follow the rule of reciprocity in their interactions will develop a mutually beneficial relationship characterized by trust and commitment (Blau, 1964; Cropanzano & Mitchell, 2005; Homans, 1961). Therefore, if exchange relationships do not entail reciprocal benefits for the people involved, they will not endure.

Negative gossip exposes individuals who misbehave in the eyes of others and warns others about social targets who might not reciprocate in social exchanges, or who perform below group standards (Beersma & Van Kleef, 2011, 2012; Feinberg, Willer, & Schultz, 2014; Feinberg et al., 2012; Sommerfeld, Krambeck, Semmann, & Milinski, 2007; Sommerfeld, Krambeck, & Milinski, 2008; Wu et al., 2016a, 2016b). Therefore, negative gossip about oneself leads to a more negative reputation in the group, which limits one’s ability to form or maintain trusting exchange relations with others, and may even threaten one’s group membership (Feinberg et al., 2014; Nowak & Sigmund, 2005). For example, a study on gossip in everyday life showed that negative gossip influences how targets are evaluated, making them less likely to be helped by others, and more likely to be avoided (Dores Cruz et al., 2020). Similarly, experimental studies demonstrated that people tend to cooperate less with negative gossip targets (Sommerfeld et al., 2007) and may ostracize them unless they conform to group norms (Feinberg et al., 2014). Ostracism has been equated with “social death” (Williams, 2007) because ostracized individuals do not benefit from their group membership and experience difficulties in fulfilling needs and goals.

Therefore, by harming targets’ reputation, negative gossip damages the extent to which they can engage in positive social exchanges with others, and has rippling negative effects on their well-being. As such, targets of negative gossip may perceive a lack of goodwill from others toward them. According to the reciprocity principle (Blau, 1964), negative gossip targets may be dissuaded from extending their goodwill to others, and may reduce their unmonitored, discretionary behaviors aimed at helping and supporting others. Hence, in line with previous research showing that negative gossip is detrimental for targets’ engagement in OCB (Kong, 2018; Wu et al., 2018a; Ye et al., 2019), we predict:

**Hypothesis 1:** Negative gossip is negatively associated with OCB of gossip targets.

### The Mediating Role of Social Inclusion

Negative gossip conveys unfavorable information, which not only harms target’s reputation and exchange relationships with others but also informs them that their attributes or behavior are not accepted in the group. It is essential for humans to feel included in groups because group membership is a central aspect of the self-concept (Baumeister & Leary, 1995) and satisfies individuals’ material and psychological needs (e.g., need for belongingness and positive self-regard). Social inclusion reflects the extent to which the group satisfies one’s fundamental needs for *belongingness* and *authenticity* (Deci & Ryan, 2000; Jansen et al., 2014). Social inclusion indicates whether the group is willing to include the self (Ellemers & Jetten, 2013), and is distinct from related constructs, like social identification, representing the emotional value individuals attach to their relationship with the group (Postmes, Haslam, & Jans, 2013), or affective commitment, representing an individual’s emotional attachment to an organization (Meyer, Allen, & Smith, 1993). In short, identification and commitment are indicators of how the individual connects to the group, assuming primary agency of the individual, whereas social inclusion reflects how the group treats the individual. The extent to which individuals perceive to be socially included is based on inclusionary signals sent by group members. Some of these signals stem from direct interactions with others (Ellemers & Jetten, 2013), but individuals are also likely to pick up on inclusionary signals from interactions they are not part of, such as gossip that targets them (Martinescu, Janssen, & Nijstad, 2019a). Awareness of negative gossip about the self may constitute a social cue that is likely to reduce one’s experienced inclusion in a group, by thwarting needs for belonging and authenticity.

First, negative gossip is likely to harm individuals’ perceptions that they belong to the group. The need to belong is satisfied by a stable bond with the group, based on recurrent and affectionate interactions (Baumeister & Leary, 1995). Negative gossip criticizes and exposes individuals’ character flaws or misbehavior. By damaging their reputation, negative gossip harms targets’ bond with the group because it lowers the likelihood of positive interactions with others (Foster, 2004; Wu et al., 2018b). People prefer to cooperate with trustworthy individuals, who demonstrate they adhere to similar values and norms (Turner, Hogg, Oakes, Reicher, & Wetherell, 1987). However, negative gossip describes targets as individuals who do no not adhere to group values and norms (Feinberg et al., 2012). Negative gossip draws a barrier between gossipers, who are in the in-group, and the target, who is in the out-group (Bosson, Johnson, Niederhoffer, & Swann, 2006; Peters, Jetten, Radova, Austin, 2017). Thus, targets hearing negative gossip are likely to learn that they are seen as perpetrators, who threaten the interests of the group, and who do not belong in the inner circle of the group. As such, negative gossip harms targets’ perception that they have a stable and affectionate bond with the group.

Furthermore, targets of negative gossip may perceive that group members are hostile to them, signaling indirectly that they do not belong in the group. Negative gossip can be used to denigrate targets and implicitly promote oneself (McAndrew, Bell, & Garcia, 2007), in a manner similar to trash talking (Kniffin & Palacio, 2018), because it can provide competitive advantages in winning resources or status at the expense of the target. In their discourse, gossipers imply that they fulfill certain standards, whereas the targets do not (Wert & Salovey, 2004). Negative gossip may also express indirect aggression toward targets (Archer & Coyne, 2005; Beersma & Van Kleef, 2012; Stirling, 1956). Thus, negative gossip targets may perceive that they do not belong with group members who harm them in order to draw personal benefits, or who are hostile and aggressive toward them.

Second, negative gossip is likely to make targets feel less socially included because they experience low authenticity in the group. The need for authenticity is satisfied when individuals’ behavior is an expression of their own choice, in accordance to their integrated sense of self (Deci & Ryan, 2000), and when they are allowed to be different from other group members (Jansen et al., 2014). Because negative gossip involves criticism of targets who do not conform to group norms (Feinberg et al., 2012; Sommerfeld et al., 2007, 2008), negative gossip conveys a signal that the group does not allow the target to be authentic. Furthermore, negative gossip exerts social control over targets, who face ostracism unless they conform to group norms (Feinberg et al., 2014; Wu et al., 2016a, 2016b). Targets cannot express their own point of view, or challenge what is said because they are absent when gossip is (initially) communicated (Foster, 2004). As such, negative gossip denies targets the opportunity to defend themselves before their reputation and relationships with the group are damaged. For example, an experimental study showed that negative gossip made targets angrier than the same information delivered directly to the person in the form of negative feedback (Martinescu et al., 2019a), presumably because gossip targets have limited opportunities for self-expression—they cannot intervene during gossip and control its consequences. As such, targets are likely to feel that their need for authenticity is not satisfied in a group where negative gossip pressures them to conform to norms, with no chance to present their own perspective in a timely manner, before reputational damage may occur.

In sum, negative gossip targets are likely to experience that their fellow group members do not see them as valued in-group members, and do not allow them to behave in authentic ways. Furthermore, people are likely to feel less socially close to group members who signal that they do not accept them (Aron et al., 1992). Targets may feel that they are seen as a threat that needs to be mitigated, rather than a valued member of the group. Negative gossip can be considered a signal that informs targets about the nature of their exchange relation with the group. Negative gossip limits the social exchanges that are possible between targets and the group to merely fulfilling contractual obligations (i.e., acting normatively), and excludes the satisfaction of individuals’ psychological need for belonging and authenticity. As such, we predict:

**Hypothesis 2:** Negative gossip is negatively related to social inclusion for targets.

Experiencing low social inclusion has severe consequences for individuals and for the groups to which they belong. People who do not feel socially included experience diminished self-regulation, reduced self-esteem, and increased distress, as the group does not fulfill their needs for belonging and authenticity (Baumeister, DeWall, Ciarocco, & Twenge, 2005; Jansen et al., 2014). In addition, low inclusion predicts more aggressive and less prosocial behavior, and lower performance (Twenge, Baumeister, DeWall, Ciarocco, & Bartels, 2007). Perceived antagonism from colleagues predicts negative work attitudes, low effectiveness, and high withdrawal (Chiaburu & Harrison, 2008). A decreased sense of inclusion makes employees less likely to adhere to organizational norms and values (Van Prooijen, van den Bos, & Wilke, 2004), or to go above and beyond their role requirements and engage in OCB, aiming to benefit their work groups. According to the reciprocity principle of SET (Blau, 1964), in groups where people feel socially included, they are likely to engage in OCB to reciprocate the benefits the group affords them, further strengthening their bond. However, negative gossip targets may decrease targets’ discretionary contributions to a group, in which satisfaction of their social inclusion needs is limited because these contributions are not likely to be reciprocated. As proposed by Ellemers and Jetten (2013), people who have inclusion needs that are not met by their group may manifest higher hostility and lower group loyalty, potentially in the form of lower OCB. Similarly, lonely employees, who are less likely to have positive exchange relationships with colleagues, engage less in OCB (Lam & Lau, 2012). Hence, we predict:

**Hypothesis 3:** Social inclusion is positively related to OCB.

Therefore, we expect that negative gossip harms targets’ experiences of being socially included in the group. Due to experiencing low inclusion, targets of negative gossip are likely to prioritize their own goals and needs over those of their group members, as they may not benefit from their investments in the group (Pearce & Randel, 2004). Because low social inclusion impairs engagement in OCB, we expect that negative gossip subsequently decreases targets’ engagement in OCB. Formally, we predict:

**Hypothesis 4:** Social inclusion mediates the negative relationship between negative gossip and OCB.

## Overview of Studies

We conducted three complementary studies with different designs, samples, and construct operationalizations. Study 1 was a correlational study in which Dutch employees reported the frequency of perceived negative gossip about themselves, their social inclusion, and OCB. Study 2 was a laboratory experiment that randomly allocated student participants to a gossip target or control condition. We measured social inclusion as a mediator variable. Furthermore, because the experimental setting might be too short-lived for participants to fully experience social inclusion in their experimental group as they would in an ongoing organizational group, we also measured social closeness with group members, which is more suitable in an experimental setting. In the laboratory study, we operationalized OCB as voluntary unmonitored donations to group members, fitting the nature of the study. Finally, Study 3 was an online scenario experiment, in which employees recruited worldwide were asked to imagine a scenario taking place in their current work setting. They were randomly allocated to a gossip target, gossip receiver, or no gossip condition, and answered measures of social inclusion and OCB.

## Study 1

### Participants and Procedure

Using an online questionnaire, employees were recruited through snowball sampling, using the network of the researchers. The survey was accessed by 647 people, 60.9 % were women, with a mean age of 36.83 years (*SD* = 13.10). Of these, 563 participants provided usable responses. Participants worked in different organizations, in 15 different sectors according to the International Standard Industrial Classification, such as health (20%), education (14%), finance (10%), or public governance (7%). Participants were asked about how they experience interactions with colleagues and their perceptions of workplace gossip about them. To ensure that work was an important part of their life and workplace gossip would be seen as meaningful, respondents were eligible if they worked at least 20 hours per week in an organization consisting of at least four persons. Because one’s power position may influence gossip perceptions (targets’ power may buffer reputational threats, Galinsky, Magee, Gruenfeld, Whitson, & Liljenquist, 2008); respondents were eligible if they did not hold a supervisory position. The final sample size was 536 respondents.^1^

### Measures

Unless mentioned otherwise, all measures were assessed using a 5-point Likert scale ranging from 1 (*never*) to 5 (*very often*), or 1 (*strongly disagree*) to 5 (*strongly agree*). Due to time constraints during data collection, for all measures below, we selected a subset of items for each scale (selecting the items with the high factor loadings in earlier studies).

#### Negative gossip

Previous studies show that negative gossip can be motivated by different intentions, such as to harm the target (McAndrew et al., 2007; Stirling, 1956) or to protect group members against targets who violate norms (Feinberg et al., 2012).^2^ Thus, to provide a robust test of our prediction that negative gossip is related to low inclusion, we operationalized negative gossip in two ways. We adapted items from the Motives to Gossip Questionnaire (Beersma & Van Kleef, 2012) to measure the **frequency of negative prosocial gossip** (3 items, e.g., “In your absence, how often do you think your direct colleagues talk about you to warn each other against your behavior?”; α =.78) and the **frequency of negative harmful gossip** (3 items, e.g., “In your absence, how often do you think your direct colleagues talk about you to put you in a negative light?”; α =.85; overall α for all negative gossip items was .87). Because these measures were highly correlated, they were treated as underlying dimensions of a negative gossip factor (see factor analysis results below).

**Social inclusion** was measured with eight items (Jansen et al., 2014; overall α = .91), representing the subdimensions of belonging (e.g., “My team/direct work environment gives me the feeling I belong”; α = .86) and authenticity (e.g., “[…] allows me to be who I am”; α = .90).

**OCB** was assessed with three items measuring behaviors directed at other individuals (Williams & Anderson, 1991, for example, “I help others who have a heavy workload”; α = .78).

#### Control variables

In the analyses, we controlled for participants’ gender because women have been shown to react more strongly than men to negative gossip (Martinescu, Janssen, & Nijstad, 2014). We also controlled for age and contract status (tenured/fixed term) because older and tenured employees have been shown to experience higher well-being at work (Scheibe & Zacher, 2013; Schumann & Kuchinke, 2020), and therefore, negative gossip might affect them less strongly. Means, standard deviations, and correlations for variables in Study 1 are presented in Table 1.

**Table 1.** Means, Standard Deviations, and Correlations for Variables in Study 1.

Variable	Mean	*SD*	1	2	3	4	5	6
1. Age	37.21	13.09	1					
2. Gender	1.60	.49	.01	1				
3. Contract	1.26	.43	−.43***	.05	1			
4. Negative gossip	1.63	.58	−.04	−.16**	−.02	1		
5. Social inclusion	3.88	.62	−.05	.11^*^	−.03	−.41***	1	
6. OCB	3.87	.59	−.15***	.21***	.04	−.14**	.27***	1

*Note. N* = 534. Gender coded 1 for men and 2 for women. Contract coded 1 for permanent and 2 for fixed. *p < .05; ** *p* < .01; *p* < .001. OCB = organizational citizenship behavior.

### Results

#### Confirmatory factor analysis

We assessed the factor structure of our measures in a confirmatory factor analysis. Items were permitted to load only on the factors they were expected to indicate, and no errors were correlated. As expected, negative prosocial gossip and negative harmful gossip were highly correlated (*r* = .62, *p* < .001). We tested a model specifying the second-order factor negative gossip consisting of negative prosocial gossip and negative harmful gossip, the second-order factor social inclusion containing the first-order factors belonging and authenticity, the two subdimensions of the construct (Jansen et al., 2014), and the first-order factor OCB. This model showed acceptable fit (χ^2^ = 684.45, *p* <. 001, *df* = 112, χ^2^/df = 6.11, root mean square error of approximation (RMSEA) = .09, comparative fit index (CFI) = .90, normed fit index (NFI) = .88, Tucker–Lewis Index (TLI) = .87, and standardized root mean square residual (SRMR) = .05).

#### Common method variance analysis

Next, we investigated the presence of common method variance by adding an unmeasured latent factor (Podsakoff et al., 2003) to our measurement model. The latent factor significantly improved model fit (Δχ^2^ = 383.71, Δ*df* = 17, *p* < .01), which implies that some common method variance influenced the validity of the factor structure. Afterward, we performed an equal constraints test to determine whether the common method variance was evenly distributed across factors. This was not the case. To account for the presence of unevenly distributed common method variance, we used bias-corrected factor scores in the analyses described below.

#### Hypotheses testing

**Figure 1.**
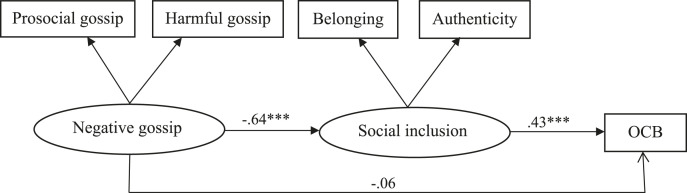
Structural equations for model 2 in study 1 (unstandardized coefficients; paths from control variables not shown).

We conducted structural equation modeling using Amos version 25 (Arbuckle, 2017) to test the hypotheses (see Figure 1). To test Hypothesis 1, stating that negative gossip is negatively related to OCB of gossip targets, we estimated Model 1 with a direct path from negative gossip (as second-order factor) to OCB, controlling for participants’ gender, age, and contract status. This model provided sufficient fit to the data (χ^2^ = 14.69, *df* = 3 χ^2^/*df* = 4.90, *p* = .002, RMSEA = .09, CFI = .99, NFI = .98, TLI= .93, and SRMR = .03). Supporting Hypothesis 1, there was a significant negative relationship between negative gossip and OCB (*b* = −.14, *p* = .02).

To test Hypothesis 2, 3, and 4, we added social inclusion (as second-order factor) in Model 2, and specified the indirect effect of negative gossip on OCB through social inclusion (see Figure 1). The indirect effect was estimated with 5000 bootstrap samples. The model approached the conventional threshold of acceptable fit (χ^2^ = 65.04, *df* = 9, χ^2^/*df* = 7.23, *p* < .001, RMSEA = .11, CFI = .97, NFI = .96, TLI = .90, and SRMR = .08). There was no direct effect of negative gossip on OCB (*b* = −.06, *p* = .26). As Hypotheses 2 and 3 predicted, negative gossip was negatively related to social inclusion (*b* = −.64, *p* < .001) and social inclusion was positively related to OCB (*b* = .43, *p* < .001). The indirect effect of negative gossip on OCB through social inclusion (*b* = −.27, 95% CI [−.41, −.16]) provides support for Hypothesis 4.^3^

### Discussion

As hypothesized, results showed that negative gossip influenced targets’ OCB, and this was mediated by their experience of social inclusion in the group. While an advantage of the results obtained in this study is that they reflect perceptions of employees regarding gossip experiences they had at their workplace, the correlational design limits drawing conclusions about the direction of the causal relationships between gossip and target’s behavior. Negative gossip may decrease citizenship behaviors for targets, but it is also plausible that ill adapted employees become the targets of negative gossip. To investigate the causal relationships between negative gossip and targets’ social inclusion and OCB, we conducted an experiment.

### Study 2

#### Participants and design

Eighty-five students at a Dutch university (*M*_age_ = 21.61, *SD*_age_ = 3.17; 53 women) participated in a laboratory experiment in exchange for course credit or 4 Euros. Participants were assigned to teams of three (16 teams were completed by a confederate, which was possible because participants did not interact). Participants were randomly assigned to a gossip condition or to a control (no gossip) condition. To observe a medium size effect with two groups at power .80 and α = .05, about 90 participants were needed (GPOWER; Erdfelder, Faul, & Buchner, 1996).

#### Procedure

Participants arrived at the laboratory for a study on verbal ability and group performance. Participants were identified by the colors blue, green, and red and were seated together in a room where the experimenter explained they would engage in three tasks: word building, sentence building, and letter search. For the word-building task, the participants remained in the same room and each person built as many words as possible from 15 scrabble letters assigned to them. They were instructed not to talk with others during this task, and that they would have an opportunity to communicate later during the experiment.

Next, participants went to individual cubicles where they engaged in the second task. After they finished, the computer informed them that they could now communicate with one or both team members if they wished to do so, by writing them a note, which would be delivered by the experimenter in the next step. Participants could use paper with the header “personal note,” on which they had to specify to whom it was addressed and who it was from. The experimenter picked up the sentences and personal notes (if any were written). Next, each participant received a set of sentences allegedly written by one of the other group members which were to be used for the third task (counting letters). All participants received the same set of 10 handwritten sentences. A fake “personal note” was among the sentences. Afterward, in order to measure participants’ voluntary prosocial behaviors directed at other group members (an operationalization of OCB that fits the nature of the experiment), they played one round of a dictator game (see also Beersma & Van Kleef, 2011; Piazza & Bering, 2008; Wu et al., 2016b). Participants read that they were randomly chosen from their group and won 15 lottery tickets, each representing a chance to win a 15 Euro prize. Participants were told that they could share any number of tickets with their group members if they wanted to, and that their decision would remain private. Next, participants filled in the measures described below were debriefed and thanked for participation.

#### Gossip manipulation

The “personal note” contained either gossip about themselves or a neutral message. In the gossip condition the note said: “Hey there, [Blue/Green/Red (color receiver)]! What do you think about [color target participant]? Didn't do much at scrabble… I saw we found a lot more words. It's not fair that we do more work for the team than [color target participant]". In the control condition the note said: “Hey there, [Blue/Green/Red (color receiver)]! This is an interesting experiment! What do you think?"

#### Measures

**Social inclusion** (α = .91) was measured as in Study 1, with 8 items. Furthermore, given the experimental setup, where participants had little time to fully develop an explicit sense of social inclusion, we also measured **social closeness** with group members, which is similar to social inclusion, because it reflects perceptions of belongingness with others and has been shown to reliably predict relationship quality (Aron, Aron, & Smollan, 1992) and is more suitable in a short-lived experimental setting. Participants indicated on a graphic item depicting inclusion of other in the self (Aron et al., 1992), how close they felt with their group members by choosing between 7 images of two circles that overlap in different degrees (see Appendix). Low overlap, scored with 1, indicated low closeness, and high overlap, scored with 7, indicated high closeness. To fit the nature of our laboratory experiment, **OCB** was operationalized as the number of lottery tickets participants allocated to their group members in the dictator game. Before finishing, participants were asked whether they had any remarks about the study, what they thought the experiment was about, and what the received note was about (manipulation check), in a series of open-ended items.

### Results

#### Participant screening and manipulation check

Five participants were excluded due to procedural and human errors (e.g., computer error, experimenter delivered the wrong notes, and participant rushed the experiment). We excluded 10 participants who believed the note was addressed to them (whereas all notes were addressed to the third group member) and 8 participants who indicated that the note was fake. The final sample consisted of 62 participants. Of these, 43 filled in an open item manipulation check^4^ and all answers matched their experimental condition (e.g., gossip condition: “they think they did all the work and I didn’t do a lot” and control condition: “she thinks it's an interesting experiment”). Means, standard deviations, and correlations for variables in Study 2 are presented in Table 2.

**Table 2.** Means, Standard Deviations, and Correlations for Variables in Study 2.

	Mean (*SD*)	1.	2.	3.	4.	5.	6.	7.	8.
1. Age	21.60 (3.20)	—							
2. Gender	1.65 (.48)	.15	—						
3. Condition	.00 (1.00)	−.005	0	—					
4. Social closeness	3.27 (1.56)	.10	−.09	−.37**	—				
5. Social inclusion	3.94 (1.06)	.02	−.13	−.40**	.32*	(.91)			
6. Tickets self	8.34 (4.02)	−.07	−.22	.12	−.28*	−.10	—		
7. Tickets to note receiver (OCB)	3.56 (2.16)	.12	.21	.004	.15	.02	−.94**	—	
8. Tickets to note sender (OCB)	3.16 (2.08)	.09	.22	−.20	.37**	.12	−.93**	.76**	—

*Note*. *N* = 62. Cronbach’s alphas are reported on the diagonal. Condition was coded with −1 for control and 1 for negative gossip. Gender coded 1 for men and 2 for women. * *p* < .05; ** *p* < .01; *p* < .001. OCB = organizational citizenship behavior.

### Hypotheses Tests

#### OCB

**Figure 2.**
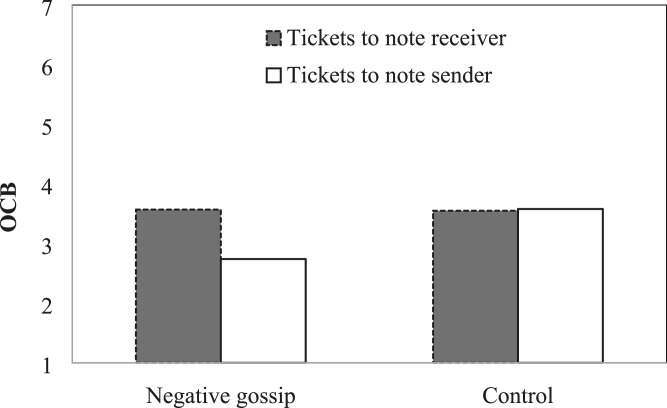
Organizational citizenship behavior as a function of gossip condition and gossip role in study 2.

We tested a mixed-model ANOVA with gossip condition as a between-subjects factor and ticket allocations to one’s group members (i.e., OCB) as a within-subjects factor (see Table 3). Results showed that participants allocated marginally fewer tickets to the sender than to the receiver of the note (*F* (1, 59) = 3.59, *p* = .06), and this effect was qualified by an interaction with the experimental condition (*F* (1, 59) = 4.29, *p* = .04). As shown in Figure 2, gossip targets allocated fewer tickets to the sender of the note (*M* = 2.74, *SD* = 2.16) than the receiver (*M* = 3.57, *SD* = 2.37), whereas in the control condition participants allocated a similar number of tickets to the sender (*M* = 3.58, *SD* = 1.94) and receiver (*M* = 3.55, *SD* = 1.98). These results are in line with Hypothesis 1.

**Table 3.** Mixed-model ANOVA Results in Study 2.

	Mean square	*F* (*df*)	*p*-value
Condition	4.05	.51 (1, 59)	.48
OCB (tickets group members)	3.75	3.59 (1, 59)	.063
Condition * OCB	4.47	4.29 (1, 59)	.043

*Note*. *N* = 62. Condition was coded with −1 for control and 1 for negative gossip. OCB = organizational citizenship behavior.

#### Social inclusion and social closeness

As predicted by Hypothesis 2, participants in the gossip target condition felt less socially included (*M* = 3.51; *SD* = .91) than participants in the control condition (*M* = 4.35; *SD* = 1.05; *t* (60) = 3.37, *p* < .001). Furthermore, gossip targets felt less socially close to their group members (*M* = 2.71; *SD* = 1.39) than participants in the control condition (*M* = 3.84; *SD* = 1.53; *t* (60) = 3.03, *p* < .01).

#### Mediation

To test Hypothesis 4, predicting that gossip decreases OCB due to lower social inclusion (and social closeness), we performed three multiple mediation analyses. Gossip condition was entered as independent variable, whereas social inclusion and closeness were entered as parallel mediators. The outcome measures (tested in three separate analyses) were ticket allocations to the self, to note sender, and to note receiver. First, social inclusion was unrelated to ticket allocation to oneself (*b* = .08, ns), but social closeness was negatively related to selfish allocations (*b* = −.73, *p* = .05) and mediated the effect of gossip condition on ticket allocation to self (*b* = .41, 95% CI [.0005; 1.06]), such that gossip targets allocated more tickets to themselves than participants in the control condition because they felt less close to their group members. Second, social inclusion did not predict ticket allocation to the note sender (*b* = −.02), although this was expected. However, in line with Hypothesis 3, perceived closeness predicted higher allocations to note sender (*b* = .45, *p* = .01) and mediated the effect of gossip condition on ticket allocation to sender (*b* = −.25. 95% CI [−.59; −.04]), such that gossip targets felt less close to their group than participants in the control condition, which led them to allocate fewer tickets to note senders. This indirect effect is in line with our expectation that the way targets feel in the group predicts their OCB. A third analysis showed that neither social inclusion nor closeness were related to ticket allocations to the note receiver (all *b* = < .26, all *p* > .19).

Although social inclusion was not related to OCB (ticket allocations to the group members; predicted by Hypothesis 3), results showed that negative gossip decreased closeness to group members, which led to higher selfish allocations and lower contributions to the group member who sent the note. As such, these findings largely support Hypothesis 4.

### Discussion

In line with our hypotheses, results showed that gossip targets engaged in OCB to a lower extent than participants in the control condition, by allocating fewer lottery tickets to the sender of the gossip note and more to themselves, and this was explained by targets’ lower social closeness with group members. Therefore, this study experimentally showed that because gossip made participants feel less close (thus less socially connected) to their group members, they chose to allocate more lottery tickets to themselves and fewer to the note sender. Findings of Study 2 are complementary to the results of Study 1. In the next study, we aimed to replicate these findings with a different sample, using a methodology that enhances external validity, and includes a different measure of OCB. As such, we conducted a scenario experiment set in participants’ workplace, and asked them to indicate how they would react to a situation (involving gossip or not).

### Study 3

#### Participants

The aim of this study was to compare employee’s responses in 3 scenarios that take place at their work (scenarios are described in the manipulation section below). An a priori power analysis indicated that a sample size of 590 is required for finding a medium–small effect at 90% power; α = .05. We invited 590 people to participate using the online platform Prolific. The survey took about 10 minutes, and participants were paid 1 GBP. To access the survey, participants had to be employed for at least 21 h/week, and work in groups with at least two other employees. We included a straightforward attention check that asked participants to click a specific response option. Forty-two participants failed the attention check, and were excluded, as were 26 who accessed the study and quit before answering a usable part. The study was reposted until the required sample size was reached. We collected 590 complete responses and 7 almost complete responses, which were retained. The final sample size was 597, of which 372 participants were men and 218 women. Mean age was 32.32 years (*SD* = 9.40). Participants had 27 different nationalities, of which most frequent were the United Kingdom (*n* = 231), the United States (*n* = 107), Poland (*n* = 65), and Portugal (*n* = 48). The highest level of education completed was secondary education for 23 participants, high school for 81, technical or community college for 46, bachelor’s degree for 252, master’s for 167, PhD for 22, and 6 did not indicate this.

Although accessing the survey on Prolific was conditioned by being currently employed, we checked whether participants were employed; 567 confirmed that they were indeed employed, 27 had last been employed on average 3.76 months before, and 3 were never employed. The 3 who had never worked before were excluded from further analyses, but the 27 who had recently become unemployed were retained, as they were able to provide details about their most recent job. The results presented below are essentially the same if these participants are excluded. Participants worked in over 40 different domains of activity, of which the most frequent were software (*n* = 56), data processing (*n* = 42), finance and insurance (*n* = 41), and health care and social assistance (*n* = 38). Participants worked on average 38.87 h/week (*SD* = 9.61), had average tenure in the current role of 4.72 years (*SD* = 5.19), and average department or group size was 27.43 people (*SD* = 28.45).

#### Procedure

Participants were asked to think of their current job and list 3 behavioral norms that they considered essential for doing their job well. For example, participants wrote: “being focused,” “good communicator,” “collaboration,” and “being innovative.” The second of the 3 behaviors identified here was used afterward in the scenario manipulations.

#### Manipulation

Participants were asked to enter (code) names for two colleagues with whom they work frequently. Afterward, they were randomly assigned to one of 3 conditions, asking them to imagine a scenario that takes place at their work, during a usual day. In the *gossip target condition*, participants were asked to imagine that they accidentally overheard the two colleagues talk about them in a negative way. The colleagues, unaware that the participant heard them, criticized the participant regarding a behavior participants identified as important in doing their job well. In *control condition 1 (gossip receiver),* participants were asked to imagine that they accidentally overheard the two colleagues talk about another coworker in a negative way. The colleagues, unaware that participants can hear them, criticized the third coworker regarding a behavior participants identified as important in doing their job well. In *control condition 2 (no gossip)*, participants were asked to imagine they are having a typical day at work with the two colleagues. To help them reflect on the scenarios, participants in all conditions were asked to write down 2–3 short sentences describing what their two colleagues may do or say. These statements were used as manipulation checks.

#### Focal measures

Unless otherwise stated, all measures presented below had a Likert scale response format, from 1 (completely disagree) to 7 (completely agree). After reading the assigned scenario, participants indicated how they would feel and behave if the scenario they read happened at their work. We measured **social inclusion** with the complete scale used in Study 1 (16 items developed by Jansen et al., 2014; α = .98). **OCB** was measured with the scale of Lee and Allen (2002), with 8 items for OCB directed at individuals (e.g., “I would assist others with their duties”; α = .90).

#### Control variables

At the start of the survey (before the manipulation), we measured job satisfaction and affective commitment to the organization, which have been documented in previous research to be stable predictors of OCB (Organ & Ryan, 1995). **Job satisfaction** was measured with 6 items (Agho, Price, & Mueller, 1992, e.g., “I find real enjoyment in my job”; α = .84). **Affective commitment** was measured with 4 items (Meyer, et al., 1993, e.g., “I feel a strong sense of belonging to my organization”; α = .94). After participants listed the 3 behavioral norms essential for their job, we measured **fulfillment of the norm** used later in the scenarios, using two items (“To what extent do you think you fulfill this requirement, in general?” and “How satisfied are you with your efforts toward this requirement?”; α = .81); the response scale for this measure was from 1 (not at all) to 7 (very much). We measured norm fulfillment since it may relate to high social inclusion levels, as people who fulfill norms satisfy inclusion criteria, as well as high OCB because they are likely to have time or resources for extra-role behaviors. Last, after the scenarios, **reputational concern** was measured with a 5-item scale from Kim and Kim (2020, e.g., “I would worry about the impression others have of me”; α = .90). We included this variable because negative gossip increases reputation concern and is likely to affect targets’ behavior in the group (e.g., Piazza & Bering, 2008). **Age** and **gender** were added as control variables as well (see rationale in Study 1). Importantly, if analyses are conducted without covariates, the statistics presented below and their interpretation are essentially the same as when the covariates are included.

At the end of the survey, a **manipulation check** item asked participants to indicate what the scenario was about by choosing one of three statements (“I overheard my colleagues talk about my behavior while they thought I did not hear them,” “I overheard my colleagues talk about someone else’s behavior while they thought I did not hear them,” and “I just had a usual day at work with my colleagues").

### Results

#### Confirmatory factor analysis (CFA)

A CFA showed that the hypothesized 8-factor structure with social inclusion as a second-order factor (comprising belonging and authenticity); OCB, job satisfaction, affective commitment, norm fulfillment, and reputational concern as separate factors fitted the data well (χ^2^ = 2672.61, *p* < .001, *df* = 762, χ^2^/*df* = 3.50, RMSEA = .07, CFI = .93, TLI = .93, and SRMR = .05).

#### Manipulation check

In the gossip target condition, 194 of 202 participants indicated that colleagues talked about them, in the gossip receiver condition, 173 of 194 indicated that colleagues talked about someone else, and in the no gossip condition, 161 of 194 indicated the scenario was about a usual work day. Although most participants recalled correctly at the end of the study the topic of the scenario, 62 of 597 participants (10.3%) indicated the wrong topic. To clarify whether these participants misunderstood the manipulation, we inspected the open answers in which they described what their colleagues may say or do in the presented scenario. The answers of 52 participants matched their condition, for example, target condition “That I'm not fulfilling the requirements,” gossip receiver condition “Colleague # 3 is fatigued and does not devote himself to his work properly,” and no gossip condition “Ask a few questions about process.” The responses of 10 participants did not match their scenario, and they were excluded from further analyses. Thus, the following analyses were based on 584 respondents. Means, standard deviations, and correlations for variables in Study 2 are presented in Table 4.

**Table 4.** Means, standard deviations, and correlations for variables in study 3.

Variable	*M* (*SD*)	1	2	3	4	5	6	7	8	9
1. Age	32.33 (9.43)	—								
2. Gender	1.38 (.50)	−.005	—							
3. Job satisfaction	4.60 (1.25)	.11**	.02	(.84)						
4. Affective commitment	4.39 (1.67)	.09*	.004	.65***	(.94)					
5. Target condition	.34 (.48)	−.05	−.05	−.03	−.02	—				
6. Norm fulfillment	5.83 (.98)	.15***	.002	.29***	.21***	−.05	(.81)			
7. Reputational concern	4.92 (1.34)	−.005	.14***	.11**	.15***	.19***	.07	(.90)		
8. Social inclusion	4.19 (1.71)	.07	0	.30***	.29***	−.38***	.14***	−.09*	(.98)	
9. OCB	5.22 (1.11)	.09*	.06	.30***	.31***	−.18***	.29***	.16***	.46***	(.90)

*Note*. *N* = 584; Cronbach’s alphas on diagonal. Target condition coded 1 for target and 0 for other.

Gender coded 1 for men and 2 for women. * *p* < .05; ** *p* < .01; *p* < .001. OCB = organizational citizenship behavior

### Hypotheses Testing

We conducted mediation analyses using the SPSS PROCESS macro. We tested whether the experimental condition predicted the mediator social inclusion, and whether social inclusion predicted OCB. Because condition is a multi-categorical variable, we used dummy coding, comparing the target condition to each of the two control conditions.

Hypothesis 1 predicted that negative gossip is associated with lower OCB for targets. Regression coefficients of the total effects showed that OCB was lower for gossip targets (*M* = 4.94, *SD* = 1.22) than gossip receivers (*M* = 5.22, *SD* = 1.03; *b* = .25, *p* = .02), and compared to people not exposed to gossip (*M* = 5.49, *SD* = .98; *b* = .62, *p* < .001). These results support Hypothesis 1.

As shown in Table 5, participants experienced lower social inclusion in the gossip target condition (*M* = 3.28, *SD* = 1.61) than the gossip receiver condition (*M* = 3.99, *SD* = 1.62; *b* = .66, *p* < .001) and the no gossip condition (*M* = 5.32, *SD* = 1.17; *b* = 2.01, *p* < .001). This is in line with Hypothesis 2, predicting a negative relation between gossip and social inclusion of gossip targets. As predicted by Hypothesis 3, social inclusion was positively related to OCB (*b* = .25, *p* < .001).

**Table 5.** Summary of Mediation Analyses for Study 3.

	Mediator social inclusion	Dependent variable OCB
	*b* (*SE*)	*b* (*SE*)
Constant	1.13 (.46)*	1.51 (.31)***
Age	.005 (.006)	.002 (.004)
Gender	−.13 (.12)	.06 (.08)
Job satisfaction	.25 (.06)***	.04 (.04)*
Affective commitment	.15 (.05)***	.07(.03)*
Norm fulfillment	.06 (.06)	.21 (.04)***
Reputation concern	−.003 (.05)	.14 (.03)***
**D1** ^a^ **: Target versus receiver**	.66 (.14)***	.08 (.10)
**D2** ^a^ **: Target versus no gossip**	2.01 (.15)***	.12 (.11)
**Social inclusion**		.25 (.03)***
		Indirect effect [95% confidence interval]
**D1:**		.16 [.08; .26]
**D2:**		.50 [.35; .66]

*Note*. *N* = 580; standard errors in parentheses; * *p* < .05; ** *p* < .01; *** *p* < .001. OCB = organizational citizenship behavior.

^a^Because the experimental condition is a multi-categorical variable, two dummy variables were used in the analysis (D1 and D2), with the target gossip condition as a reference category. For D1, target condition was coded with 0, receiver with 1, and no gossip with 0; for D2, target and receiver were coded with 0 and no gossip with 1. The two dummies were entered in the analysis simultaneously.

Furthermore, targets compared to gossip receivers indicated they would engage in less OCB due to lower social inclusion (*b* = .16, 95% CI [.08; .26]), as did targets compared to people hearing no gossip (*b* = .50, 95% CI [.35; .66]). These results support Hypothesis 4.

### Discussion

Findings of Study 3 are consistent with results from Studies 1 and 2, and provided further support for our predictions, showing that negative gossip targets felt less socially included than receivers of gossip about other people, or those who heard no gossip. Moreover, due to lower social inclusion, targets indicated they would engage in OCB less than participants in the other conditions. This pattern was observed when controlling for other predictors of OCB and for reputation concern, a well-documented effect of negative gossip (Piazza & Bering, 2008; Wu et al., 2016a). This supports predictions that social inclusion is a distinct process that explains gossip targets’ behavioral intentions toward group members.

## General Discussion

Understanding the effect of negative gossip on the OCB of targeted individuals is of considerable social importance, given that well-functioning groups rely on their members to contribute voluntarily, without constant monitoring (Nielsen et al., 2009). Our research yielded two clear findings. First, this research shows that negative gossip is causally related to low OCB of gossip targets. Second, this research focuses on a novel mechanism through which gossip decreases targets’ OCB. In all three studies, negative gossip targets experienced low social inclusion, which was related to low OCB in Studies 1 and 3 (in Study 2, low social closeness was related with low OCB). By decreasing their social inclusion (closeness in Study 2), negative gossip indirectly led to a decrease of targets’ OCB. These results provide robust evidence of the destructive effect of negative gossip on targets’ exchange relation with the group, which negatively affects their unmonitored OCB, a key resource for groups.

### Theoretical Implications

In three studies, this research provided coherent findings about the effect of negative gossip on targets’ OCB by introducing a meaningful mediator, social inclusion. Negative gossip has been shown to stimulate targets to overtly increase contributions to the group to protect their reputation (Beersma & van Kleef, 2011; Feinberg et al., 2012, 2014; Sommerfeld et al., 2007, 2008; Wu et al., 2016a, 2016b). However, negative gossip is also related to a decrease in targets’ OCB due to a more negative self-concept (Kong, 2018; Wu et al., 2018a), lower organizational identification (Ye et al., 2019), or lower emotional resilience (Wu et al., 2018b). Our article contributes to a more a comprehensive understanding of how negative gossip affects targets’ relationship with the group and their behavior in the group, using different methodologies, samples, operationalizations, and relevant control variables. This allows for theoretical integration of inconsistent or diverging conclusions from prior research.

First, relying on SET (Blau, 1964; Cropanzano & Mitchell, 2005), this work shows that negative gossip harms the exchange relation between targets and the group, which is detrimental for targets’ group benefitting behavior. Negative gossip thwarts target’s fundamental needs for belonging and authenticity (i.e., social inclusion), and this makes targets less inclined to contribute to the group willingly, beyond minimal, contractual requirements. By damaging targets’ perception of the group as willing to socially include them, negative gossip subsequently decreases targets’ OCB. Therefore, this study shows that by damaging targets’ social inclusion, negative gossip disrupts the positive reciprocity loops that people tend to develop over time with the groups they are part of.

Second, our work demonstrates that negative gossip has causal effects on OCB and social inclusion of targets. These results are consistent with findings of studies using organizational samples, which document an association between negative gossip and OCB (Kong, 2018; Wu et al., 2018a; Ye et al., 2019). However, our work addresses an important limitation of correlational studies: in Studies 2 and 3, experimental manipulations of negative gossip demonstrated that negative gossip determined target’s low OCB compared to behavior of control groups. Furthermore, Study 1 documented a negative association between negative gossip and social inclusion, and Studies 2 and 3 showed that negative gossip was causally related to targets’ low social inclusion. As such, our work demonstrates that being the target of negative gossip is related to lower feelings of social inclusion and becoming less generous, helpful, and supportive toward group members (i.e., OCB).

Third, this article employed a multi-method approach for testing the mediated effect of negative gossip on targets’ OCB through social inclusion. We conducted three studies with different samples, designs, and construct operationalizations. Study 1 was a correlational study among Dutch employees, Study 2 was an experiment conducted among Dutch students, and Study 3 was an experiment among employees recruited around the world (of 27 different nationalities) and used vignettes set in their current work context. Negative gossip was measured differently in each study. In Study 1, participants indicated how frequently group members gossip about them in general; in Study 2, one group member unilaterally gossiped about the target; and in Study 3, participants pictured negative gossip exchanged between two colleagues they often work with. Our research reliably showed that regardless of how negative gossip was operationalized, it had a negative effect on social inclusion and OCB. Moreover, different operationalizations of OCB were used: different scales for self-rated OCB in Studies 1 and 3 and observed discretionary cooperative behavior in Study 2. This multi-method approach provides high internal and external validity to the findings.

Fourth, the mediating effect of social inclusion was observed when including theoretically meaningful covariates. In Study 3, participants’ reputation concern was especially relevant as control variable because reputation is one of the core mechanisms that make gossip effective in influencing targets’ behavior (Feinberg et al., 2012; Wu et al., 2016a, 2016b). As such, it can be expected that negative gossip targets engage in desirable behavior to manage their reputation. However, as shown in Study 3, negative gossip had a negative effect on OCB due to lower social inclusion, above and beyond its effect on OCB through reputation concern. This suggests that negative gossip sets in motion multiple psychological mechanisms, which explain unique variance in targets’ OCB. Moreover, for demonstrating the incremental validity of our propositions about the mediating role of social inclusion, in Study 3, we included job satisfaction and affective commitment as covariates because these constructs have been shown to positively predict OCB (Organ & Ryan, 1995). Controlling for these factors allowed us to demonstrate that social inclusion explains unique variance in OCB.

### Practical Implications

Our results are of particular importance from an applied point of view, given that gossip is part of everyday life (Dunbar et al., 1997), and people are likely to overhear negative gossip about themselves. Findings of this research indicate that targets of negative gossip decrease their OCB because they perceive their group members as hostile and unwilling to socially include them. By reducing OCB, negative gossip harms the interests of the group and of the organization. Therefore, managers should implement strategies to limit negative gossip and to mitigate its’ detrimental effects. Drawing from our results, interventions should address the mechanisms related to gossip targets’ experiences in the group. Approaching targets and communicating openly about the problems at hand and offering genuine social support might be helpful in mitigating low inclusion. People respond with less anger and retaliation to negative feedback than to negative gossip, possibly because feedback offers targets the opportunity to defend or repair their image, whereas gossip does not (Martinescu et al., 2019a). Furthermore, because targets who feel responsible for the negative gossip are likely to engage in repairing behavior (Martinescu et al., 2019a), disseminating the gossip incident in a supportive environment may help targets reflect on the gossip, analyze the critique, and strive to repair their mistakes. However, such approaches are likely to be time consuming and to require emotional labor from all parties.

In addition to preventing or mitigating effects of negative gossip on social inclusion and OCB, organizations could focus on strengthening individuals’ perceptions of being socially included. Managers could create activities and design work that foster positive bonds between employees, such as team building, coaching, and decreasing competitive incentives. Furthermore, organizations could endorse values and norms that encourage acceptance of authentic behavior. Employees who experience high social inclusion are likely to have stable, positive exchange relations with colleagues, making them less vulnerable to negative gossip.

### Limitations and Future Research

Our research has some noteworthy limitations. First, in Study 1, the data were collected from the same source (employees) at one point in time, which raises concerns about common method bias and inferring causality. Although this type of data captures employees’ real life experiences, providing external validity to the findings, a downside is the likelihood of common method bias. However, this was accounted for in our analyses. Moreover, to alleviate concerns about common method bias, in Studies 2 and 3, gossip was experimentally manipulated rather than assessed by participants, and findings were consistent across the 3 studies. Furthermore, the experimental designs of Studies 2 and 3 allowed us to draw conclusions about causal effects of negative gossip on social inclusion and OCB. Future research should consider adding longitudinal designs to the set of studies presented here, in order to test the effects of negative gossip on social inclusion and OCB over a longer time.

Second, in Studies 1 and 3, we used self-report measures of OCB, which could be problematic because socially desirable responses might bias reports about own engagement in OCB. Other-rated measures of OCB have been preferred in previous research (Xie et al., 2019; Ye et al., 2019). However, a meta-analysis showed that self-reported measures are suitable for measuring OCB (Carpenter, Berry, & Houston, 2014). We used self-reported measures because we were interested in OCB that is unmonitored by colleagues or managers. Furthermore, to mitigate concerns about social desirability in self-reports, in Study 2, we used a behavioral measure of OCB (i.e., private donation to group members).

Third, some of the results reported in Study 2 did not strictly support the hypotheses. That is, the measure of social inclusion was not related with OCB in this study. As outlined in the introduction to Study 2, it is likely that in an experimental setting, participants have little opportunity to fully develop a clear sense of social inclusion. For this reason, we additionally measured social closeness, which fits the experimental context better than the generic inclusion measure. Results showed that social closeness predicted OCB in the expected way.

Fourth, it is important to note that boundary conditions could exacerbate or reduce the effects we documented, and future studies should investigate these. For example, the frequency of being a negative gossip target may determine how targets perceive the gossip. Although our studies show that isolated gossip incidents already influence social inclusion and OCB, they may be less harmful than constantly being a gossip target. Furthermore, despite its negative effects, some negative gossip may help targets gain insights about their own behavior, their position in the social network, or relationships with others. For example, research shows that direct insults received from rivals motivate people to increase their effort and perform better (Yip, Schweitzer, & Nurmohamed, 2018). Similarly, future studies could identify if there is a “tipping point,” determining when gossip may be functional for targets and when it has predominantly negative effects. For example, targets’ power position may buffer the effect of negative gossip on social inclusion, especially if gossipers have low power and targets have high power (Galinsky et al., 2008). Conversely, when target’s peers gossip negatively with their superiors, the effect on social inclusion may be stronger because upward gossip may be especially damaging for targets (Martinescu, Janssen, & Nijstad, 2019b). Thus, future research should investigate if boundary conditions moderate the effects of negative gossip for targets.

Fifth, our research focused on social inclusion as determined exclusively by negative gossip. However, in reality, multiple factors are likely to influence individuals’ sense of social inclusion, for example, social cues given over time by group members during direct interactions (Ellemers & Jetten, 2013). Thus, it is plausible that targets of negative gossip who have a high pre-existing level of social inclusion are more resilient to negative gossip than those experiencing low inclusion because their exchange relationship with the group may be less vulnerable to negative gossip. It remains for future research to test this possibility.

Last, this study focused exclusively on the effects of negative gossip. However, because positive gossip is omnipresent and is often intertwined with negative gossip (Kniffin & Wilson, 2005), it would be interesting for future research to investigate how positive and mixed gossip may influence targets’ experience of social inclusion and OCB.

## Concluding Remarks

Our results showed that targets of negative gossip experience low social inclusion, which decreases their OCB. This study helps bridge two previously disconnected lines of research examining the effect of negative gossip on targets’ cooperative behaviors through different psychological mechanisms (e.g., reputation concern and social identification). As such, in line with field studies conducted in organizations (Kong, 2018; Wu et al., 2018a; Ye et al., 2019), our findings showed that due to its detrimental impact on targets’ social inclusion, negative gossip may be a less effective way of enabling sustainable cooperation in groups than recent experimental studies claim it to be (cf. Wu et al., 2016a, 2016b). Further research is needed in order to understand the effects of negative gossip on group viability in the long term, and the complex mechanisms through which these effects come about. We hope research will continue to explore the effects of gossip on all involved parties in order to increase understanding of the implications of this omnipresent phenomenon in organizations.

## Appendix

### Measure of Social Closeness Used in Study 2

We are interested in how you perceive the similarities and differences between yourself and your team members. Please tick one pair of circles that best represents the similarities and differences between yourself and your team members. Y = you and T = your team members.

## Notes

1 Testing the hypotheses by analyzing the responses of the 563 participants, who provided usable answers, with multiple regression yielded similar conclusions as tests reported below.

2 Gossip senders and receivers (of gossip about others) distinguish between negative gossip driven by these two motives (Beersma & Van Kleef, 2012): hearing gossip about other people that is prosocially motivated (aimed at helping the receiver) is perceived to be morally justified, acceptable, and less socially undesirable than negative gossip intended to harm the target (Beersma & Van Kleef, 2012). However, we expected that targets of gossip would perceive both types of gossip as damaging for them, as from the target’s perspective, it matters less whether the negative gossip is driven by prosocial intentions or by gossiper’s intention to harm them. Because all gossip targets are vulnerable to reputational damage, regardless of the gossiper’s motive, negative gossip is likely to be experienced by its targets as threatening, harming their exchange relationships (Foster, 2004; Wu et al., 2018b). We thus expected that targets would experience reduced social inclusion in response to both types of negative gossip about them because all negative gossip, regardless of gossipers' motive, implies they are not included in the group.

3 Similar indirect effects were obtained for models in which negative harmful gossip and negative prosocial gossip were modeled as separate independent variables.

4 Participants were first asked if they received a note. Nineteen participants indicated that they did not, which is not surprising in hindsight, as the note was not addressed to them. We then presented the participants who answered that they did receive a note with the open item manipulation check (*n* = 43).
